# The influence of the Kinesio Taping on selected ultrasonography measurements, and quality of life in patients with rotator cuff lesions

**DOI:** 10.1038/s41598-020-75701-6

**Published:** 2020-10-29

**Authors:** Aneta Bac, Magdalena Wróbel, Katarzyna Ogrodzka-Ciechanowicz, Edyta Michalik, Anna Ścisłowska-Czarnecka

**Affiliations:** Faculty of Motor Rehabilitation, The Bronisław Czech University of Physical Education, al. Jana Pawła II 78, 31-571 Kraków, Poland

**Keywords:** Diseases, Health care

## Abstract

The assessment of the six-week influence of Kinesio Taping combined with a rehabilitation on selected ultrasonography measurements, the level of disability, and the quality of life in patients with rotator cuff lesions. 60 participants were randomly assigned into a taping group (KT combined with a six-week rehabilitating protocol) and a control group (only rehabilitation protocol). In all patients the following assessments were performed twice: USG, UEFI and NHP questionnaires. In the examination of the subacromial space and the subacromial bursa in the taping group, no statistical significance was observed. A statistically significant change in the thickness of the muscles was obtained only for the thickness of the infraspinatus in the taping group. A statistically significant change was obtained in the assessment of tendinopathy only for the supraspinatus muscle in both groups. Within both groups a statistically significant difference was observed in the average UEFI and NHP scores; however, the differences in the scores obtained between the groups were not statistically significant. The use of KT with a rehabilitation program did not yield statistically significantly better results in the improvement of selected shoulder region indicators, the function of the upper limb and the quality of life.

## Introduction

Damage to the rotator cuff is one of the most common causes of shoulder joint pain, and its prevalence increases with age. This affliction is more common in the dominant hand, and it affects men more often^1^. Full-thickness damage is found in approximately 25% of people over 60 years of age, and the risk factors include previous shoulder injuries, diabetes, smoking, hypercholesterolemia, and genetic factors^2–6^.

A rapid progression of this condition results in subacromial impingement syndrome, tendinopathy, calcific tendinitis, tears, bursitis, and bursal reactions. In physical examination, patients suffering from rotator cuff injuries complain of chronic shoulder pain and weaker flexion, abduction, and external rotation, which significantly impairs daily activities^7^.

In recent years, Kinesio Taping (KT) has become a very popular method of treating locomotor diseases. According to research, this method aids and accelerates the rehabilitation process for various types of muscle or joint injuries^1^. KT also improves microcirculation and increases lymph flow, which in turn promotes tissue regeneration within the affected areas. Depending on the technique of tape application, KT normalizes muscle tone and has an analgesic effect^8^.

There are reports in the available literature on the effectiveness of KT in the treatment of shoulder joint diseases^9–11^. The vast majority of these studies examine the effect of KT on pain, range of motion, or strength of the rotator cuff muscles, while only some studies check the effectiveness of KT in improving the functional performance of the upper limb. However, there are no reports on the impact of KT on the quality of life of patients, or on the actual effect of KT in healing injured muscles or reducing inflammation, as evaluated by imaging tests such as MRI or ultrasound. In addition, a large proportion of the studies describe the effectiveness of short-term therapy on small groups of patients and often assess the effectiveness of KT without a concurrent rehabilitation program.

Compared to other imaging techniques, ultrasound is an inexpensive real-time test. It enables the assessment of the rotator cuff during dynamic examination of the shoulder joint, in comparison with the opposite shoulder as well. It is also tolerated well by patients^6,12^. USG allows for diagnosis of the extent of rotator cuff tendon damage (partial or complete) and for visualization of the locus of damage. It is the basis for the diagnosis of shoulder joint pathologies, and is a method for monitoring the effectiveness of therapy^12^. Research indicates that ultrasound has the same accuracy and sensitivity as MRI in the diagnosis of full-thickness tears, but is less sensitive than MRI in detecting partial tears^6^.

According to our best knowledge, in the current literature there is no scientific evidence describing the long-term effects of Kinesio taping (KT) on the functional parameters of the upper extremity, assessed with ultrasonography, and the quality of life in patients with shoulder dysfunction, so the aim of this study was to assess the six-week influence of KT combined with a rehabilitation program in comparison to a rehabilitation program alone on selected ultrasonography measurements, the level of disability, and the quality of life in patients with rotator cuff lesions.

## Materials

Sixty patients qualified for the study. The participants were assigned randomly by the first author to an experimental group and a control group. Qualification was based on simple randomization (a coin toss) and was carried out by the main author. Patients assigned to the taping group underwent a rehabilitation protocol that included Kinesio taping. The control group underwent the same protocol, excluding Kinesio taping. Eventually, there were two groups of 30 patients each; the patients ranged in age from 25 to 60 years.

There were 32 women (53.3%) and 28 men (46.7%) in the study. In the taping group there were 15 women and 15 men (50% each), and in the control group there were 17 women (56.7%) and 13 men (43.3%). Sex was not a significant differentiating factor in our study (*p* = 0.604). The specific characteristics of the groups are outlined in Table 1.

**Table 1:** Characteristics of the participants.

	N	$\overline{x}$	Me	Min	Max	Q1	Q3	SD	*p*
**Age [years]**									
Taping group	30	45.67	49.00	25.00	60.00	37.00	52.00	9.29	0.273
Control group	30	48.17	50.50	28.00	60.00	45.00	56.00	10.35	
Total	60	46.92	49.00	25.00	60.00	38.50	55.00	9.83	
**Height [cm]**									
Taping group	30	171.07	170.50	158.00	185.00	164.00	176.00	7.71	0.109
Control group	30	167.83	166.50	155.00	187.00	163.00	172.00	7.71	
Total	60	169.45	170.00	155.00	187.00	164.00	175.50	7.81	
**Weight [kg]**									
Taping group	30	82.43	81.50	53.00	128.00	71.00	90.00	17.27	0.540
Control group	30	79.87	78.00	55.00	105.00	72.00	91.00	14.97	
Total	60	81.15	80.00	53.00	128.00	71.50	90.50	16.07	

Inclusion criteria:

- between 20 and 60 years of age
- subacromial bursitis
- tendinopathy of at least one rotator cuff muscle
- edema in the area of attachment, tendon, or muscle belly of at least one rotator cuff muscle
- signs of subacromial impingement

Exclusion criteria:

- severe arthrosis
- complete rupture of one of the tendons of a rotator cuff muscle
- injury to other muscles surrounding the shoulder (except a rotator cuff)
- shoulder ligament lesions
- surgery of the shoulder
- previous injuries to the glenohumeral joint
- injuries to the muscles surrounding the shoulder joint
- neuromuscular abnormalities
- skin diseases
- medications which affect the musculoskeletal system, i.e., trimetafan

Patients informed and written consent was obtained, and the rights of subjects were protected.

The research project obtained the approval of the Ethical Committee of the Regional Medical Chamber in Krakow (No. 41/KBL/OIL/2015).

The measurements were carried out at the University of Physical Education in Krakow in the Faculty of Motor Rehabilitation, in collaboration with the Dyga Med Outpatient Rehabilitation Clinic.

This study is registered in the Australian and New Zealand Clinical Trials Registry, Registration no.: ACTRN12617000624381 (date of registration: 01.05.2017).

## Methods

### Rehabilitation program

#### Rehabilitation protocol

The rehabilitation program lasted 6 weeks and consisted of 3 55-min sessions per week. The program was identical for both the taping and control groups. Each session was conducted one-to-one with the same physical therapist. Each session consisted of 3 parts:

The first phase (approx. 5 min.) consisted of warm-up exercises to prepare the tissues for more intense exercises. This phase included free active exercises (of low intensity) of the upper extremities and trunk in closed kinematic chains.

The main phase (approx. 35 min) consisted of exercises in the lying, sitting, and standing positions. The protocol included isometric, active exercises without any external force applied, active exercises with external force, sensomotoric exercises, and Proprioceptive Neuromuscular Facilitation (PNF) exercises. This protocol led to the activation of all muscles in the shoulder girdle.

The last phase (approx. 5 min.) consisted of stretching and relaxing exercises.

#### Methodology of KT

Patients from the taping group underwent the standard rehabilitation protocol in conjunction with KT. The tape was applied to relieve tendons that had lesions. All of the patients in this group had tape applied to their deltoid muscle. The tape was changed every 4 days for a period of 6 weeks. The first application of tape was done right after the first examination of the patient.

Application on deltoid muscles:

- The technique of applying a V-shaped tape over the muscle was used.
- The base of the tape was applied on the muscle attachment—the deltoid tuberosity—applied in a neutral position.
- The upper limb in the shoulder joint was flexed and in internal rotation—in this position the back part of the tape was applied without tension, towards the spine of the scapula.
- The patient then rotated the limb internally—in this position the anterior part of the tape was glued, in the direction of the acromion^13^.

Application on supraspinatus muscles:

- The technique of applying an “I” tape to the muscle was used.
- The base was applied at the head of the humerus—the upper part of the greater tubercle—with the upper limb in active abduction to about 20°.
- The limb was returned to a neutral position, and the end of the tape was applied without tension towards the supraspinous fossa^13^.

Application on infraspinatus muscles:

- The technique of applying a V-shaped tape to the muscle was used.
- The base of the tape was applied to the greater tubercle, in a neutral position.
- The upper limb was set in protraction with internal rotation.
- Two strips of tape were applied so that the upper part was under the spine of the scapula and the lower part covered the inferior angle of the scapula. The tape was applied without tension^13^.

Application on subscapularis muscles:

- The same technique was used as for the infraspinatus muscles, except that the upper part of the tape was applied above the superior angle of the scapula^13^.

Application on teres minor muscles:

- The muscle technique was used for applying tape in the shape of the letter “I”.
- The upper limb was set in abduction up to 90° in internal rotation. In this position the tape base was applied to the lower part of the greater tubercle.
- The tape was then applied without tension along the dorsal surface of the lateral border of the scapula^13^.

### Assessment tools

In all individuals participating in the project (taping and control groups), the following assessments were performed twice (always before and after the rehabilitation program):

- USG.
- UEFI questionnaire to assess functionality in the upper extremities.
- NHP questionnaire to assess health-related quality of life.

All questionnaires were administered by the same physiotherapist, and the ultrasound examination was performed by the same orthopedic physician. All of the tests were carried out at the same time of day (in the morning).

Shoulder ultrasound was performed with a certified Esaote MyLabSeven ultrasound machine. To assess the shoulder area, a 13-MHz linear transducer was used. The goal of the ultrasound diagnosis was to determine the type and size of damage to the rotator cuff. The examination evaluated the following:

- subacromial space (measured in mm)
- subacromial bursa in position 0 (measured in mm)
- bursa in internal rotation (measured in mm)
- rotator cuff muscle thickness (measured in mm)
- impingement syndrome, − no limited range of motion (ROM) and no pain, limited ROM and pain, or no limited ROM but pain
- the presence of tendinopathy, measured as plus “+” (tendinopathy affecting most of the tendon), minus “−” (no tendinopathy) or plus/minus “+/−” (tendinopathy involving less than half of the tendon).

T**he Upper Extremity Functional Index (UEFI)** is a questionnaire which assesses the extent of upper-limb disability in people with orthopedic injuries. The questionnaire contains 20 questions about the level of difficulty in performing activities of daily living. For each question, the patient reports the difficulty of an activity on a scale from 0 to 4, where 0 represents an inability to perform the activity and 4 means that the activity can be performed without difficulty. The maximum score that can be achieved is 80 points, and the lower the score, the greater the disability of the limb^15^.

**The Nottingham Health Profile (NHP)** questionnaire assesses health-related quality of life and consists of two parts. The first part focuses on current physical, psychological, and social problems related to health and measures six dimensions of health: physical mobility, pain, social isolation, emotional reactions, energy, and sleep. The second part of the questionnaire assesses the impact of health on seven areas of life: employment, household activities, social life, home life, sex life, hobbies and interests, and holidays. All of these questions have only yes/no answer options; one point is awarded for each positive answer. The higher the score, the lower the quality of life in that area^16^.

### Statistical methods

Statistical analysis of the gathered data was carried out using Statistica 10.0 (StatSoft). The following parameters were used: the means, medians, minimums, maximums, and standard deviations of the body mass, body weight, and ages of the patients. Normal values were verified with the Shapiro–Wilk test. For statistical analysis we used Student’s t-test, the Mann–Whitney U test, and the Wilcoxon test. In all of the tests, the level of significance was set at *p* < 0.05.

## Results

In the examination of the subacromial space and the subacromial bursa in a neutral position (0) and in internal rotation in the taping group, no statistical significance was observed. For the same indicators assessed in the control group, statistical significance was found only for changes in the size of the subacromial space. A comparison was made between the taping and control groups for all variables. No statistical significance was found for any of them (see Table 2).

**Table 2:** Selected indicators of the shoulder/joint region (mm) for the study groups before and after therapy.

		Taping group						Control group					
		N	x	SD	Min	Max	*p*	N	x	SD	Min	Max	*p*
Subacromial space	Before therapy	30	10.86	3.75	0.00	17.10	0.717	30	11.16	2.16	6.10	17.10	0.025
	After therapy	30	11.09	1.86	8.20	14.90		30	10.64	2.12	5.90	16.30	
	Comparison between the groups	*p* = 0.652											
Bursa in position 0	Before therapy	30	1.06	0.82	0.00	3.10	0.589	30	1.14	0.68	0.00	2.60	0.347
	After therapy	30	1.14	0.64	0.00	2.60		30	1.02	0.70	0.00	2.90	
	Comparison between the groups	*p* = 0.297											
Bursa in internal rotation	Before therapy	30	1.27	0.87	0.00	3.10	0.380	30	1.17	0.70	0.00	2.90	0.548
	After therapy	30	1.39	0.64	0.00	2.60		30	1.14	0.83	0.00	3.90	
	Comparison between the groups	*p* = 0.280											

The next indicator to be examined was subacromial impingement symptom. For this symptom, no statistically significant differences were noted in either group before and after therapy (see Table 3).

**Table 3:** Subacromial impingement symptom in the study groups before and after therapy.

Impingement symptom		Taping group			Control group		
		N	%	*p*	N	%	*p*
Before therapy	No limited ROM, no pain	12	40	0.050	15	50	0.172
	Limited ROM, pain	15	50		12	40	
	No limited ROM, pain	3	10		3	10	
After therapy	No limited ROM, no pain	22	73.3		22	73.3	
	Limited ROM, pain	6	20		4	13.3	
	No limited ROM, pain	2	6.7		4	13.3	

*ROM* range of motion.

During the study, the thickness of the muscles that make up the rotator cuff was measured twice. A statistically significant change in this variable was obtained only for the thickness of the infraspinatus in the taping group. A comparison of the results of the two groups did not reveal any statistically significant differences for any of the tested muscles (see Table 4).

**Table 4:** Rotator cuff muscle thickness for the study groups before and after therapy.

Muscle thickness (mm)		Taping group						Control group					
		N	x	SD	Min	Max	*p*	N	x	SD	Min	Max	*p*
Supraspinatus SSP	Before therapy	30	5.52	1.07	3.60	8.00	0.001	30	5.21	0.84	3.80	7.10	0.904
	After therapy	30	5.09	0.90	3.20	6.80		30	5.10	0.73	3.90	7.00	
	Comparison between the groups	*p* = 0.066											
Infraspinatus ISP	Before therapy	30	4.18	0.99	2.40	6.30	0.745	30	4.30	0.73	3.00	5.80	0.967
	After therapy	30	4.16	0.96	2.30	6.40		30	4.33	0.61	3.20	5.40	
	Comparison between the groups	*p* = 0.678											
Teres minor TM	Before therapy	30	2.18	0.70	1.10	3.70	0.052	30	2.21	0.56	1.20	3.10	0.050
	After therapy	30	1.99	0.51	1.30	3.30		30	2.03	0.40	1.30	2.90	
	Comparison between the groups	*p* = 0.911											
Subscapularis SSC	Before therapy	30	3.71	1.02	1.50	5.90	0.484	30	3.71	1.06	2.00	6.40	0.297
	After therapy	30	3.75	0.89	1.40	5.50		30	3.83	0.96	2.00	5.80	
	Comparison between the groups	*p* = 0.700											

Another pathology examined in this study was tendinopathy, which was evaluated for all the muscles that make up the rotator cuff. A statistically significant change was obtained in the assessment of this symptom only for the supraspinatus muscle in both the taping group and the control group (see Table 5).

**Table 5:** Symptom of rotator cuff muscle tendinopathy for both study groups.

Tendinopathy			Taping group			Control group		
			N	%	*p*	N	%	*p*
Supraspinatus (SSP)	Before therapy	Plus ( +)	14	46.7	0.036	17	56.7	0.020
		Minus (−)	7	23.3		4	13.3	
		Plus/minus (+ /−)	9	30		9	30	
	After therapy	Plus ( +)	7	23.3		10	33.3	
		Minus (−)	8	26.7		6	20	
		Plus/minus (+ /−)	15	50		14	46.7	
	Comparison between the groups	Before therapy vs. After therapy	*p* = 0.066					
Infraspinatus (ISP)	Before therapy	Plus ( +)	6	20	0.529	5	16.7	0.179
		Minus (−)	20	66.7		24	80	
		Plus/minus (+ /−)	4	13.3		1	3.3	
	After therapy	Plus ( +)	3	10		3	10	
		Minus (−)	24	80		25	83.3	
		Plus/minus (+ /−)	3	10		2	6.7	
	Comparison between the groups	Before therapy vs. After therapy	*p* = 0.678					
Teres minor (TM)	Before therapy	Plus ( +)	0	0	0.179	2	6.7	0.150
		Minus (−)	28	93.3		27	90	
		Plus/minus (+ /−)	2	6.7		1	3.3	
	After therapy	Plus ( +)	0	0		1	3.3	
		Minus (−)	30	100		27	90	
		Plus/minus (+ /−)	0	0		2	6.7	
	Comparison between the groups	Before therapy vs. After therapy	*p* = 0.911					
Subscapularis (SSC)	Before therapy	Plus ( +)	5	16.7	0.767	7	23.3	0.176
		Minus (−)	17	56.7		18	60	
		Plus/minus (+ /−)	8	26.7		5	16.7	
	After therapy	Plus ( +)	2	6.7		3	10	
		Minus (−)	22	73.3		21	70	
		Plus/minus (+ /−)	6	20		6	20	
	Comparison between the groups	Before therapy vs. After therapy	*p* = 0.700					

During the study, rotator cuff muscle damage was also assessed. Damage to only the supraspinatus muscle was observed in two people in the taping group and in two people in the control group before therapy; these values did not change after therapy for either of the study groups.

The degree of disability of the upper limb in the studied patients was measured using the UEFI questionnaire. Within both groups (taping and control), a statistically significant difference was observed in the average scores obtained by patients from both groups; however, the differences in the scores obtained between the groups were not statistically significant (see Table 6).

**Table 6:** Assessment of upper-limb disability using the UEFI questionnaire for the two study groups.

UEFI	Taping group						Control group					
	N	x	SD	Min	Max	*p*	N	x	SD	Min	Max	*p*
Before therapy	30	50.83	14.03	17.00	75.00	0.001	30	52.40	13.09	21.00	70.00	0.001
After therapy	30	66.63	10.91	39.00	80.00		30	65.73	11.02	34.00	80.00	
Comparison between the groups	*p* = 0.151											

All subjects were assessed for health-related quality of life using the NHP questionnaire. The findings showed that a statistically significant improvement in quality of life was obtained in both the taping and control groups, but a comparison of the results of the two groups did not show such a relationship (see Table 7).

**Table 7:** Quality of life, as evaluated by the NHP questionnaire for the study groups.

NHP		Taping group						Control group					
		N	x	SD	Min	Max	*p*	N	x	SD	Min	Max	*p*
“Yes” response	Before therapy	30	12.30	6.97	2.00	30.00	0.001	30	13.37	7.65	3.00	31.00	0.002
	After therapy	30	7.60	8.07	0.00	30.00		30	9.10	7.13	0.00	29.00	
	Comparison between the groups	*p* = 0.318											
“No” response	Before therapy	30	32.57	7.23	15.00	43.00	0.001	30	31.73	7.84	14.00	42.00	0.002
	After therapy	30	36.50	9.81	7.00	45.00		30	35.93	7.16	16.00	45.00	
	Comparison between the groups	*p* = 0.297											

## Discussion

The proper functioning of the upper limbs in everyday activities is one of the basic aspects of a high quality of life. Disability in even one upper limb translates into difficulties performing basic everyday activities, and can also lead to restrictions in practicing one’s profession. However, despite the many modern forms of rehabilitation, functional recovery of the upper limbs is a long process and does not always bring the expected results, which is why physiotherapists are constantly looking for new solutions that would help improve the effectiveness of treatment for shoulder injuries^17^. Our study has shown that the use of CT in conjunction with a rehabilitation program did not yield statistically significantly better results in the improvement of selected shoulder region indicators assessed by ultrasound than the rehabilitation program itself.

KT is an auxiliary therapeutic method which consists in applying elastic tape to the affected muscles; it combines physiotherapy with the naturally occurring process of self-healing. KT is a modern and universal method that can be used to treat many different diseases and joint/muscle injuries, by reducing pain and swelling and improving circulation and muscle and joint function^13,14^.

The available literature discusses various methods of conservatively treating rotator cuff tears, while there are no studies delineating the effect of KT on the size of the subacromial space, the subacromial impingement syndrome and tendinopathy, or the thickness of the tendons of the rotator cuff muscles and the volume of the subacromial bursa, as assessed by means of imaging tests such as USG. This is important because such a study could determine whether KT can be helpful, not only in the treatment of pain or swelling, but also in improving the range of mobility in the joint. To the best knowledge of the authors of this study, this project is the first attempt to assess the long-term effect of KT combined with a rehabilitation program on the above-mentioned indicators in patients with rotator cuff injury. A single report in the literature by Kaya et al.^18^ describes the effect of KT only on the thickness of the suprascapular muscle tendon diagnosed by ultrasound. In their research, the authors compared KT combined with physical exercises to KT combined with manual therapy. After six weeks of observation, it was observed that both therapies had a similar effect on the tendons of patients with subacromial impingement symptom. Our study evaluated a number of indicators describing the soft tissues of the shoulder area in patients with rotator cuff injury. It was noted that after the 6-week therapy in the group, USG imaging showed a statistically significant increase in the number of patients without subacromial impingement, as well as a statistically significant change in the thickness of the supraspinate muscle; in the control group, the only statistically significant results were the change in the size of the subacromial space and the reduction of supraspinatus tendinopathy. Although we observed these changes, the comparison of all the indicators included in the study before and after therapy revealed that the results of the two groups do not differ in a statistically significant way.

The available literature includes publications measuring the impact of KT on the degree of disability of the upper extremity, though the outcomes of these studies are ambiguous. The reason behind this may be that researchers use different tools to assess the functionality of the upper limbs, the time of intervention varies considerably, and the effectiveness of KT is compared to other different therapeutic methods. One of the researchers who attempted to evaluate the degree of disability is Kaya et al.^19^. In their study, they used the DASH questionnaire to assess the impact of Kinesio Taping compared to the effect of physical therapy in patients with shoulder impingement syndrome. The results showed that after two weeks of therapy, the study groups did not differ in a statistically significant way. Further studies conducted by Kaya et al.^18^ assessed the effect of KT in combination with exercises versus KT combined with manual therapy. The six-week therapy included 54 patients with subacromial impingement syndrome, and the DASH questionnaire was used to measure therapeutic progress. There was no statistically significant difference in the degree of upper limb disability in the two groups.

Similar results were obtained in a study by Thelen et al.^1^, who examined 42 patients with rotator cuff muscle inflammation. The degree of disability of the upper limb was assessed with the SPADI questionnaire, and the observation time was six days. The results did not indicate any statistically significant differences between before the therapy and afterwards. Frassanito et al.^10^ checked the effectiveness of shock wave treatment in combination with KT compared to shock wave treatment by itself. A total of 42 people with rotator cuff tendinopathy were qualified for the study. Patients from the treatment group got three shock wave treatments (once a week), after which KT was applied on the muscles of the rotator cuff. To assess the degree of disability of the upper limb, researchers used DASH, the Subjective Shoulder Rating Questionnaire (SSRQ), and the Oxford Shoulder Score (OSS). The results indicated a higher effectiveness of KT therapy in improving upper limb function according to each questionnaire. Also, based on the study of Simsek et al.^9^, KT combined with physical therapy provides statistically significantly better results than the exercises themselves. The researchers drew their conclusions based on a 12-day therapy program used in 38 patients with subacromial impingement syndrome. Kul and Ugur^20^ came to opposite conclusions, comparing the effectiveness of KT combined with home exercises to classic physiotherapy combined with home exercises. The study involved 40 patients with shoulder impingement syndrome, and upper limb disability was assessed using the Western Ontario Rotator Cuff assessment (WORC) after 15 days of therapy. The findings demonstrated better results in the group of patients to whom KT was not administered. Our research confirms the reports by Kaya et al.^18,19^. UEFI questionnaire was used to assess upper limb disability in 60 patients with rotator cuff injury. After the 6-week therapy program, no statistically significant differences were found between the groups in the reduction of upper extremity disability.

One of the important indicators assessing the effectiveness of the proposed therapy is the quality of life of the patients. Quality of life is very important in relation to the functioning of upper limbs, because this part of the body plays a role in everyday life and virtually every activity. Upper-limb impairment reduces the patient’s mobility and self-sufficiency, so when planning therapy we should also look for the opportunity to use therapeutic techniques that could further improve its performance. There are no publications that have assessed the effectiveness of using KT in improving the quality of life of patients with various disorders of the shoulder area, which seems to be a large gap in the literature due to the growing popularity of this method, as well as the increasing percentage of patients with shoulder disorders. Our research undertakes to bridge this gap. We assessed the impact of KT combined with a 6-week rehabilitation program in relation to the rehabilitation program itself on the quality of life of patients. The assessment tool was the NHP questionnaire. The applied therapy resulted in an improvement in the quality of life of patients in both the treatment and control groups by decreasing the average scores of “Yes” answers obtained in the NHP questionnaire and increasing the average number of “No” answers on the NHP questionnaire; these results were statistically significant. However, a comparison of the results obtained before and after therapy for both groups showed that the effectiveness of KT from the rehabilitation program is the same as of the rehabilitation program itself. Such findings suggest that, as in the case of other indicators assessed in our research, KT seems to have no impact on improving the quality of life.

## Conclusions

Our study has shown that the use of CT in conjunction with a rehabilitation program did not yield statistically significantly better results in the improvement of selected shoulder region indicators assessed by ultrasound than the rehabilitation program itself. Furthermore, the use of KT with a rehabilitation program brought comparable results to the rehabilitation program itself in terms of improving the function of the upper limb and the quality of life of the patients.

### Study limitation

This study is not without limitations. An important aspect that could affect trial results is a relatively small number of people in the study groups. This sample size could be a cause of e.g. the potential lack of power in differentiating between groups based on the UEFI and NHP.
